# Early changes in immunoglobulin G levels during immune checkpoint inhibitor treatment are associated with survival in hepatocellular carcinoma patients

**DOI:** 10.1371/journal.pone.0282680

**Published:** 2023-04-07

**Authors:** Lorenz Balcar, David Bauer, Katharina Pomej, Tobias Meischl, Mattias Mandorfer, Thomas Reiberger, Michael Trauner, Bernhard Scheiner, Matthias Pinter

**Affiliations:** 1 Department of Internal Medicine III, Division of Gastroenterology and Hepatology, Medical University of Vienna, Vienna, Austria; 2 Liver Cancer (HCC) Study Group Vienna, Medical University of Vienna, Vienna, Austria; 3 3^rd^ Medical Department (Hematology & Oncology), Hanusch Krankenhaus, Vienna, Austria; University of Oxford, UNITED KINGDOM

## Abstract

**Background & aims:**

Immunotherapy represents the new standard of care in systemic first-line treatment of hepatocellular carcinoma (HCC). Biomarkers that predict treatment response and survival remain an unmet clinical need.

**Methods:**

Patients with HCC treated with immune-checkpoint inhibitors (ICI) between 10/2017 and 03/2022 were retrospectively evaluated. Immunoglobulin levels (IgG, IgM, IgA) were measured at baseline and six weeks after initiation of ICI treatment. Impact of relative changes on overall survival (OS), progression-free survival (PFS), and time to progression (TTP) were evaluated.

**Results:**

Seventy-two patients with HCC receiving ICI (mostly atezolizumab/bevacizumab n = 54,75%) were included (mean age: 68±12 years, cirrhosis: 72%, mean Child-Turcotte-Pugh [CTP] score: 7±2 points). Most patients had a preserved performance status (ECOG-PS 0, n = 45, 63%), 25 (35%) showed macrovascular invasion, and 32 (44%) had extrahepatic spread.

Baseline immunoglobulin values (median, IgG: 1395mg/dL, IgM: 337mg/dL, IgA: 89mg/dL) were not different between responders and non-responders, and neither baseline nor follow-up immunoglobulin values correlated with OS, PFS, and TTP. However, the relative change in IgG (Δ-IgG) independently predicted OS in multivariable Cox regression analysis after adjusting for severity of liver disease, baseline AFP and CRP as well as for Δ-IgA and Δ-IgM. Patients could be stratified into high (Δ-IgG≥+14%) vs. low (Δ-IgG<+14%) risk groups (median OS: 6.4 vs. 15.9 months; p = 0.001). Importantly, Δ-IgG was also associated with PFS and TTP on adjusted multivariable Cox regression analyses.

**Conclusion:**

Our study proposes a higher increase of Δ-IgG upon ICI treatment as a negative prognostic marker in patients with HCC, independent of underlying liver disease severity. These results require independent validation.

## Introduction

Hepatocellular carcinoma (HCC) represents the most common primary liver cancer with poor prognosis in advanced stages, where systemic therapy is indicated [1]. Only recently, the immune checkpoint inhibitor (ICI)-based combination of atezolizumab plus bevacizumab replaced tyrosine kinase inhibitors (i.e., sorafenib and lenvatinib) as standard of care in systemic front-line [2–4]. The dual ICI regimen of durvalumab and tremelimumab is expected to be approved shortly as an alternative in first-line [3], after having improved its primary endpoint overall survival (OS) versus sorafenib in a phase III trial [5].

Despite these recent advances, there are no broadly accepted biomarkers for clinical decision making in HCC patients undergoing immunotherapy [6]. Potential tools including the immune subclasses [7], inflammatory gene signatures [8], or the recently proposed CRAFITY score [9] require validation in prospective studies but support the investigation of inflammation markers as predictors of immunotherapy efficacy.

In patients with melanoma, plasma cells expressing immunoglobulin A (IgA) and G (IgG) have been associated with poor prognosis [10]. Results from a small prospective study strengthened the potential role of immunoglobulins as predictors of survival in melanoma patients [11].

In this retrospective single-centre study, we evaluated the association of immunoglobulins with survival in patients with advanced HCC treated with ICI-based therapies.

## Methods

### Study design

Patients with HCC treated with ICI-based regimens between October 2017 and March 2022 at the Department of Internal Medicine III, Division of Gastroenterology and Hepatology of the Vienna General Hospital/Medical University of Vienna were retrospectively evaluated. According to the European Association for the Study of the Liver (EASL) guidelines [3], HCC was diagnosed by histology or dynamic imaging (computed tomography [CT] or magnetic resonance imaging [MRI] scans). Serum immunoglobulin levels were measured at immunotherapy initiation (baseline) and 6 weeks thereafter. Delta (Δ)-immunoglobulin changes were calculated by the percent change from baseline values.

### Patients and definitions

Of ninety-one patients treated with ICIs, nineteen patients with missing baseline immunoglobulin values were excluded. Information on clinical course and laboratory parameters were collected from the clinical documentation system. Liver function was assessed by Child-Turcotte-Pugh (CTP) score and tumour stage was classified according to the Barcelona Clinic Liver Cancer (BCLC) staging system [12]. Patients who had at least 1 radiological follow-up imaging assessment were evaluated for best radiological response according to the modified Response Evaluation Criteria in Solid Tumours (mRECIST) [13]. Disease control rate (DCR) was defined as the proportion of patients achieving complete/partial response or stable disease as best radiological response. Objective response rate (ORR) was defined as the proportion of patients achieving complete/partial response as best radiological response.

### Statistics

Statistical analyses were performed using IBM SPSS Statistics 27 (SPSS Inc., Armonk, NY, USA) and GraphPad Prism 8 (GraphPad Software, La Jolla, CA, USA). Continuous variables were reported as mean ± standard deviation (SD) or median (interquartile range [IQR]), and categorical variables were shown as numbers (n) and proportions (%) of patients. Comparisons of proportions and of continuous variables were performed by chi-squared test and Student’s t-test/ANOVA. Non-normally distributed variables were compared by the Mann-Whitney- U-test or the Kruskal-Wallis test.

Estimated follow-up time was calculated by the reverse Kaplan-Meier method. Initiation of immunotherapy was considered as baseline. Hence, overall survival (OS) was defined as the time from baseline until date of death or last contact and time to progression (TTP) was defined as time from baseline to first radiological progression. Patients without progression were censored at the date of last imaging. Only patients with at least one follow-up imaging were included in TTP analysis. Progression-free survival (PFS) was defined as baseline until first radiological progression or death, whatever came first. Patients who were alive/lost to follow-up without progression were censored at the date of last contact.

Potential prognostic factors were evaluated in uni- and multivariable Cox regression analyses. Only variables with p-values < 0.1 in univariable analysis were included into a multivariable Cox regression model. We have performed backward elimination for outcomes of interest to present a data-driven approach to model selection [14,15]. Survival curves were calculated using the Kaplan-Meier method and compared by log-rank tests. Based on area under the receiver operating curve (AUROC) analysis, the cut-off value with the highest sensitivity and specificity (Youden index) in identifying patients at low vs. high-risk of death for Δ-IgG was calculated. A two-sided p-value < 0.05 was considered statistically significant.

### Ethics

The study was conducted in accordance with the principles of the Declaration of Helsinki and was approved by the local ethics committee (2033/2017 und 1759/2015). The requirement of written informed consent was waived by the ethics committee.

## Results

### Patient characteristics

Of ninety-one patients treated with ICI-based therapies at our division, seventy-two were included into this study, while nineteen had to be excluded due to missing baseline immunoglobulin values (S1 Fig).

Mean age of the study cohort was 68±12 years and most patients were male (n = 51, 71%) (Table 1). Viral hepatitis B or C were the most common aetiologies of liver disease (n = 23, 32%), followed by non-alcoholic fatty liver disease (NAFLD; n = 19, 26%), alcohol-related liver disease (ARLD; n = 15, 21%), and other aetiologies of liver disease (n = 15, 21%). Mean Child-Turcotte-Pugh (CTP) score was 7±2 points; thirty-two patients (51%) had CTP class A, twenty-six patients (36%) had CTP class B, and nine patients (13%) had CTP class C. The majority had BCLC stage B (n = 16, 22%) and C (n = 45, 63%). Most patients were treated with atezolizumab/bevacizumab (n = 54, 75%), whereas twelve patients (17%) were treated with pembrolizumab, four (6%) with nivolumab and two (3%) with atezolizumab monotherapy (Table 1). Median estimated follow-up was 17.3 (7.2–27.4) months.

**Table 1:** Baseline patient characteristics.

*Patient characteristics*		Study cohort, n = 72
Age, years, mean ± SD		68.1±11.9
Sex, n (%)		
	Male	51 (71%)
	Female	21 (29%)
Aetiology, n (%)		
	Viral	23 (32%)
	NAFLD	19 (26%)
	ARLD	15 (21%)
	Other	15 (21%)
BMI, kg*m^-2^, mean ± SD		26.8±4.9
Prior surgery/ablative therapy, n (%)		39 (54%)
Prior systemic therapy, n (%)		18 (25%)
Cirrhosis, n (%)		52 (72%)
CTP score, points, mean ± SD		6.9±1.9
	A, n (%)	37 (51%)
	B, n (%)	26 (36%)
	C, n (%)	9 (13%)
Macrovascular invasion, n (%)		25 (35%)
Extrahepatic spread, n (%)		32 (44%)
ECOG PS, n (%)		
	0	45 (63%)
	1	23 (32%)
	2	4 (6%)
BCLC stages, n (%)		
	A	2 (3%)
	B	16 (22%)
	C	45 (63%)
	D	9 (13%)
Type of immunotherapy		
	Atezolizumab/Bevacizumab	56 (78%)
	Pembrolizumab	12 (17%)
	Nivolumab	4 (6%)
Laboratory parameters, median (IQR)		
	IgG, mg/dL	1395 (1035–1708)
	IgA, mg/dL	337 (248–481)
	IgM, mg/dL	89 (54–150)
	CRP, mg/dL	0.9 (0.4–2.5)
	AFP, ng/mL	87 (5–1399)

Abbreviations: AFP alpha fetoprotein; ARLD alcohol-related liver disease; BCLC Barcelona Clinic Liver Cancer; BMI body mass index; CRP C-reactive protein; CTP Child-Turcotte-Pugh score; ECOG PS Eastern Cooperative Oncology Group Performance Status; Ig immunoglobulin; IQR interquartile range; NAFLD non-alcoholic fatty liver disease; SD standard deviation.

### Impact of baseline immunoglobulins on HCC-specific outcomes

Median IgG, IgA, and IgM levels were 1395 mg/dL (IQR: 1035–1708), 337 mg/dL (IQR: 248–481), and 89 mg/dL (IQR: 54–150). There was no difference between responders (complete or partial) and non-responders in median baseline IgG (responder [R]: 1410 mg/dL vs. non-responder [non-R]: 1480 mg/dL; p = 0.752), IgA (R: 354 mg/dL vs. non-R: 337 mg/dL; p = 0.825), and IgM (R: 87 mg/dL vs. non-R: 96 mg/dL; p = 0.840) (S2 Fig). Neither baseline nor follow-up (at week six) IgG, IgA, or IgM levels were associated with OS, PFS, or TTP (S1 Table).

### Δ-IgG level is independently associated with OS

Immunoglobulin values six weeks post immunotherapy initiation were available in fifty-nine patients. Of thirteen excluded patients, six patients died prior to the follow-up measurement, two patients had a follow-up shorter than six weeks, and five patients had missing immunoglobulin levels at week six. Median Δ-IgG, Δ-IgA, and Δ-IgM were +6.2 (IQR: -1.3, +5.5)%, +10.9 (IQR: -0.5, +25.9)%, and +4.4 (IQR:-6.1, +6.1)%. The Δ of all three immunoglobulin subclasses was associated with OS (in months) in univariable Cox regression analyses (Δ-IgG: hazard ratio [HR]: 1.04 [95%CI: 1.01–1.07]; p = 0.004; Δ-IgA: HR: 1.03 [95%CI: 1.02–1.05]; p<0.001; Δ-IgM: HR: 1.01 [95%CI: 1.00–1.02]; p = 0.020) (Table 2). In multivariable analysis, only Δ-IgG (aHR: 1.04 [95%CI: 1.01–1.07]; p = 0.004) along with CRP and CTP class C remained independent predictors for OS (Table 2). In the subgroup of patients with preserved liver function at baseline (i.e., CTP A5-B7), the Δ of all immunoglobulins was associated with OS in univariable analysis but only Δ-IgG was independently associated with OS (aHR: 1.04 [95%CI: 1.00–1.08]; p = 0.030) (S2 Table).

**Table 2:** Uni- and multivariable Cox regression analyses of prognostic factors for overall survival (OS) (n = 59, events n = 38).

*Patient characteristics*		Univariable		Multivariable–first step		Multivariable–last step	
		HR (95%CI)	p-value	aHR (95%CI)	p-value	aHR (95%CI)	p-value
Age, year		1.01 (0.99–1.04)	0.299	-	-	-	-
Aetiology of liver disease							
	ARLD	1	-	-	-	-	-
	Viral	0.88 (0.35–2.23)	0.794	-	-	-	-
	NAFLD	0.84 (0.33–2.11)	0.705	-	-	-	-
	Other	0.41 (0.14–1.17)	0.096	-	-	-	-
MVI		1.00 (0.49–2.03)	0.999	-	-	-	-
EHS		0.54 (0.26–1.13)	0.100	-	-	-	-
CTP score							
	A	1	-	1	-	1	-
	B	2.01 (0.96–4.23)	0.065	1.85 (0.84–4.07)	0.126	1.72 (0.80–3.67)	0.163
	C	5.50 (2.00–15.15)	**<0.001**	5.68 (1.83–17.60)	**0.003**	5.65 (1.99–16.02)	**0.001**
ECOG PS							
	0	1	-	-	-	-	-
	≥1	1.60 (0.82–3.12)	0.166	-	-	-	-
Baseline AFP, per 1000, ng/mL		1.03 (1.00–1.05)	**0.025**	1.02 (0.99–1.05)	0.157	-	-
Baseline CRP, mg/dL		1.23 (1.09–1.38)	**<0.001**	1.21 (1.05–1.39)	**0.007**	1.23 (1.08–1.39)	**0.002**
Δ-IgG		1.04 (1.01–1.07)	**0.004**	1.04 (1.01–1.07)	**0.021**	1.04 (1.01–1.07)	**0.004**
Δ-IgA		1.03 (1.02–1.05)	**<0.001**	1.01 (0.99–1.04)	0.315	-	-
Δ-IgM		1.01 (1.00–1.02)	**0.020**	1.00 (0.99–1.01)	0.788	-	-

Abbreviations: AFP alpha fetoprotein; ARLD alcohol-related liver disease; CRP C-reactive protein; CTP Child-Turcotte-Pugh score; ECOG PS Eastern Cooperative Oncology Group Performance Status; EHS extrahepatic spread; Ig immunoglobulin; MVI macrovascular invasion; NAFLD non-alcoholic fatty liver disease

**Fig 1:**
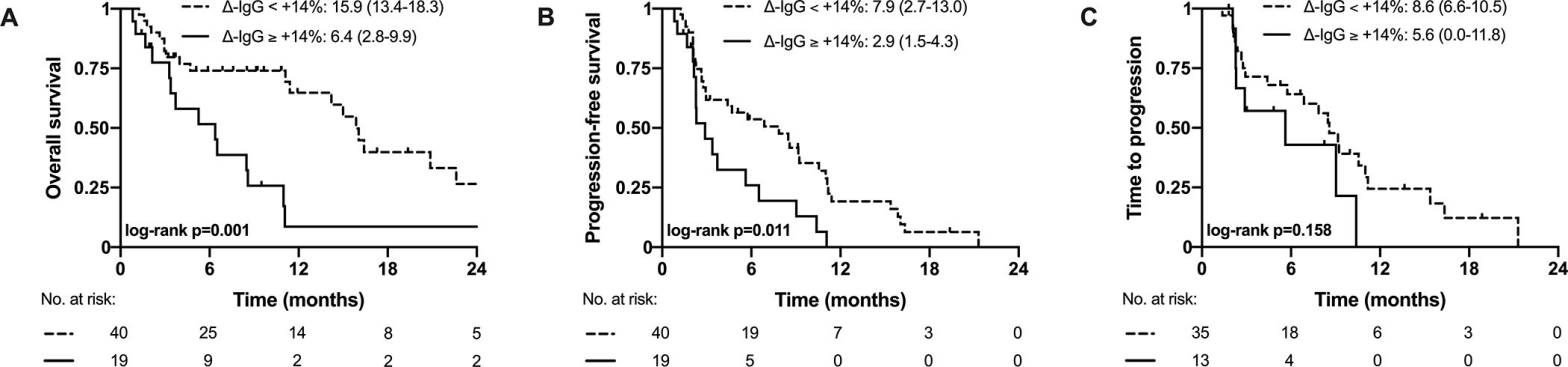
Kaplan-Meier curves for overall survival (A), progression-free survival (B), and time to progression (C) according to Youden’s optimized cut-off for percent change of Δ-IgG.

To evaluate a clinical applicable cut-off of Δ-IgG, the cut-off value with the highest sensitivity and specificity (Youden index) was +14% Δ-IgG change for OS. This cut-off separated patients with low/high risk of death (< +14%: 15.9 [95%CI: 13.4–18.3] vs. ≥ +14%: 6.4 [95%CI: 2.8–9.9] months; p = 0.001) (Fig 1A) and remained independently associated with OS in multivariable analysis (aHR: 2.45 [95%CI: 1.18–5.11]; p = 0.017) (S3 and S4 Tables). Baseline characteristics between patients with Δ-IgG < +14% ≥ +14% were similar, except of ECOG PS (S5 Table).

### Δ-IgG level is independently associated with PFS and TTP

In univariable Cox regression analysis, Δ-IgG (HR: 1.04 [95%CI: 1.01–1.06]; p = 0.004; Table 3) as well as Δ-IgM (HR: 1.01 [95%CI: 1.00–1.02]; p = 0.040) were associated with PFS, but not Δ-IgA. After adjusting for covariables, CRP, CTP class C and Δ-IgG (aHR: 1.04 [95%CI: 1.01–1.06]; p = 0.002) (Table 3) remained significantly associated with PFS in multivariable analysis. Similar results were observed in patients with preserved liver function (Δ-IgG aHR: 1.05 [95%CI: 1.02–1.09]; p = 0.003) (S6 Table). The Δ-IgG cut-off of +14% stratified patients according to PFS (< +14%: 7.9 [95%CI: 2.7–13.0] vs. ≥ +14%: 2.9 [95%CI: 1.5–4.3]; p = 0.011) (Fig 1B).

**Table 3:** Uni- and multivariable Cox regression analyses of prognostic factors for progression-free survival (PFS) (n = 59, events n = 50).

*Patient characteristics*		Univariable		Multivariable–first step		Multivariable–last step	
		HR (95%CI)	p-value	aHR (95%CI)	p-value	aHR (95%CI)	p-value
Age, year		1.00 (0.97–1.02)	0.812	-	-	-	-
Aetiology of liver disease							
	ARLD	1	-	-	-	-	-
	Viral	1.94 (0.83–4.52)	0.126	-	-	-	-
	NAFLD	1.31 (0.55–3.13)	0.544	-	-	-	-
	Other	0.83 (0.32–2.10)	0.687	-	-	-	-
MVI		0.58 (0.31–1.08)	0.084	0.56 (0.26–1.21)	0.140	-	-
EHS		1.29 (0.72–2.33)	0.394	-	-	-	-
CTP score							
	A	1	-	1	-	1	-
	B	0.95 (0.51–1.76)	0.860	1.00 (0.51–1.96)	1.000	1.09 (0.58–2.07)	0.782
	C	2.95 (1.23–7.06)	**0.015**	3.31 (1.30–8.47)	**0.012**	3.08 (1.25–7.62)	**0.015**
ECOG PS							
	0	1	-	1	-	-	-
	≥1	1.95 (1.08–3.53)	**0.027**	2.03 (0.98–4.24)	0.058	-	-
Baseline AFP, per 1000, ng/mL		1.02 (0.99–1.04)	0.194	-	-	-	-
Baseline CRP, mg/dL		1.15 (1.02–1.30)	**0.026**	1.22 (1.06–1.41)	**0.007**	1.18 (1.03–1.34)	**0.016**
Δ-IgG		1.04 (1.01–1.06)	**0.004**	1.03 (1.01–1.06)	**0.021**	1.04 (1.01–1.06)	**0.002**
Δ-IgA		1.02 (0.99–1.03)	0.092	0.99 (0.97–1.02)	0.596	-	-
Δ-IgM		1.01 (1.00–1.02)	**0.040**	1.00 (0.99–1.02)	0.600	-	-

Abbreviations: AFP alpha fetoprotein; ARLD alcohol-related liver disease; CRP C-reactive protein; CTP Child-Turcotte-Pugh score; ECOG PS Eastern Cooperative Oncology Group Performance Status; EHS extrahepatic spread Ig immunoglobulin; MVI macrovascular invasion; NAFLD non-alcoholic fatty liver disease.

Only Δ-IgG and extrahepatic spread were associated with TTP in uni- and multivariable analyses (Table 4). In patients with preserved liver function, Δ-IgG was associated with TTP in univariable but not multivariable Cox regression analyses (S7 Table). The Δ-IgG with the cut-off of +14% could not stratify patients with distinct TTP (< +14%: 8.6 [95%CI: 6.6–10.5] vs. ≥ +14%: 5.6 [95%CI: 0.0–11.8]; p = 0.158) (Fig 1C).

**Table 4:** Uni- and multivariable Cox regression analyses of prognostic factors for time to progression (TTP) (n = 48, events n = 31).

*Patient characteristics*		Univariable		Multivariable–first step		Multivariable–last step	
		HR (95%CI)	p-value	aHR (95%CI)	p-value	aHR (95%CI)	p-value
Age, year		1.01 (0.98–1.04)	0.583	-	-	-	-
Aetiology of liver disease							
	ARLD	1	-	-	-	-	-
	Viral	1.88 (0.57–6.17)	0.301	-	-	-	-
	NAFLD	1.75 (0.54–5.60)	0.349	-	-	-	-
	Other	1.37 (0.40–4.71)	0.619	-	-	-	-
MVI		0.46 (0.21–1.02)	0.057	0.63 (0.27–1.44)	0.271	-	-
EHS		2.06 (1.00–4.21)	**0.049**	2.14 (1.02–4.50)	**0.044**	2.34 (1.13–4.85)	**0.022**
CTP score							
	A	1	-	-	-	-	-
	B	0.77 (0.35–1.69)	0.520	-	-	-	-
	C	1.07 (0.24–4.73)	0.934	-	-	-	-
ECOG PS							
	0	1	-	-	-	-	-
	≥1	1.77 (0.82–3.79)	0.143	-	-	-	-
Baseline AFP, per 1000, ng/mL		1.01 (0.96–1.05)	0.799	-	-	-	-
Baseline CRP, mg/dL		1.06 (0.85–1.31)	0.613	-	-	-	-
Δ-IgG		1.03 (1.01–1.07)	**0.022**	1.04 (1.00–1.07)	**0.029**	1.04 (1.01–1.07)	**0.011**
Δ-IgA		1.01 (0.98–1.03)	0.629	-	-	-	-
Δ-IgM		1.01 (0.99–1.02)	0.384	-	-	-	-

Abbreviations: AFP alpha fetoprotein; ARLD alcohol-related liver disease; CRP C-reactive protein; CTP Child-Turcotte-Pugh score; ECOG PS Eastern Cooperative Oncology Group Performance Status; EHS extrahepatic spread; Ig immunoglobulin; MVI macrovascular invasion; NAFLD non-alcoholic fatty liver disease.

### Impact of Δ-immunoglobulins on radiological response

Median Δ-IgG (non-R: +6.3% vs. R: +5.8%; p = 0.900), Δ-IgA (non-R: +12.5% vs. R: 5.1%; p = 0.157) and Δ-IgM (non-R: +7.9% vs. R: +1.1%; p = 0.349) were not different between responders and non-responders (S3 Fig). Δ-IgG had no impact on best radiological response (S8 Table).

## Discussion

Biomarkers for risk prediction/stratification in patients with advanced HCC treated with immunotherapy are an unmet clinical need [2]. In the current study, we found that baseline and follow-up immunoglobulin levels were not predictive for survival, but percent change of IgG at week six was associated with OS, PFS, and TTP, independently of liver function and other relevant covariables.

Immunoglobulins are increasing across disease stages in advanced chronic liver disease (ACLD) [16] with an increased turnover rate being part of an immunological response [17]. However, their role in chronic liver diseases is discussed controversially. In patients with non-alcoholic fatty liver disease (NAFLD)/non-alcoholic steatohepatitis (NASH), higher IgG levels were associated with increased risks of hepatic decompensation and mortality [18], and serum IgA levels were independent predictors of advanced fibrosis [19,20]. In contrast, Basho and colleagues [21] reported that hypergammaglobulinemia was a rather protective factor in patients with decompensated ACLD, reflecting a more intact ability to sufficiently produce IgG antibodies in the light of systemic inflammation. Hence, they proposed hypergammaglobulinemia as an indicator for a preserved immune system [21]. IgG also independently predicted development of acute-on-chronic liver failure (ACLF) and short-term transplant-free survival [22]. Prognostic implications of immunoglobulins might also convert from early stages to later stages of ACLD. Since single determination of immunoglobulin levels may only predict short-term outcomes, the prognostic impact of repetitive IgG values remains a topic for future studies. To exclude severity of underlying liver disease as a confounding factor, we did not only include liver function assessed by CTP class in our multivariable models, but also performed separate analyses only in patient with preserved liver function, with similar results for Δ-IgG (S2, S5, S6 and S8 Tables). The lack of statistical significance in multivariable analysis of TTP was likely due to the low sample size in this subgroup which mitigated statistical power. In patients with preserved liver function at study initiation, 3 patients (3/40, 8%) showed a worsening of liver function (i.e., CTP>B7) at 6-week re-evaluation of immunoglobulins. Results were not different when excluding these patients.

Immunoglobulins may also play a role in malignant diseases, including HCC. In hepatitis B virus (HBV)-related HCC, IgG immunopositivity in tumour samples was associated with higher levels of core-fucosylated AFP, larger tumours, and higher prevalence of portal vein invasion [23]. In *in vitro* studies, HCC-derived IgG promoted growth of liver cancer cells [23]. In a longitudinal validation cohort, they showed that IgG levels decreased after curative surgery, but increased in patients with HCC recurrence after surgery [23]. Inflammation induced IgA positive cells were linked to immunosuppression, inhibition of an anti-tumoral cytotoxic CD8+ T lymphocyte response, and tumorigenesis in mouse models of NASH [24]. In melanoma patients treated with immunotherapy, clustering of plasma cells (mainly producing IgA and IgG) was associated with worse survival by an antigen-driven response that facilitated melanoma progression [10], and higher baseline serum IgG levels were associated with better overall survival [11].

Main limitations of our study are its retrospective design and the limited sample size. The cohort was heterogeneous in terms of liver function, treatment line, and type of immunotherapy, potentially leading to a selection bias. We also included patients with advanced or decompensated liver cirrhosis–often treated in real-life practice–and the role of immunoglobulins in cirrhotic patients is controversially discussed. While decreased values may be protective in earlier stages of liver disease [18], they seem to be negatively associated with outcomes in decompensated patients [21,22]. To avoid potential bias, we not only adjusted for disease severity, but also confirmed our results–in part–in patients with preserved liver function in separate models.

In conclusion, our findings suggest a potential role of IgG changes for outcome prediction in patients with hepatocellular carcinoma treated with immune checkpoint inhibitors. Although our results need prospective validation, our data elucidate new directions for biomarker studies in patients with advanced HCC undergoing immunotherapy.

## Supporting information

S1 Fig Patient flowchart. (TIFF)

S2 Fig Comparison of baseline immunoglobulin values according to responder and non-responder status. (TIFF)

S3 Fig Comparison of percent change of Δ-immunoglobulin values according to responder and non-responder status (ORR). (TIFF)

S1 Table Univariable Cox regression analyses of immunoglobulin levels at immunotherapy initiation and at week six on overall survival, progression-free survival, and time to progression. (DOCX)

S2 Table Uni- and multivariable Cox regression analyses of prognostic factors for overall survival (OS) in patients with preserved liver function at baseline (i.e., Child-Pugh A5-B7) (n = 40, events n = 24). (DOCX)

S3 Table Uni- and multivariable Cox regression analyses of prognostic factors for overall survival (OS) (n = 59, events n = 38). (DOCX)

S4 Table Uni- and multivariable Cox regression analyses of prognostic factors for overall survival (OS) in patients with preserved liver function at baseline (i.e., Child-Pugh A5-B7) (n = 40, events n = 24). (DOCX)

S5 Table Comparison of baseline characteristics between the Δ-IgG ≥ +14% vs. < +14% groups.
(DOCX)

S6 Table Uni- and multivariable Cox regression analyses of prognostic factors for progression-free survival (PFS) in patients with preserved liver function at baseline (i.e., Child-Pugh A5-B7) (n = 40, events n = 32). (DOCX)

S7 Table Uni- and multivariable Cox regression analyses of prognostic factors for time to progression (TTP) in patients with preserved liver function at baseline (i.e., Child-Pugh A5-B7) (n = 34, events n = 23). (DOCX)

S8 Table Association of Δ-immunoglobulin G with best radiological response. (DOCX)

S1 File (XLSX)

S2 File (PDF)

## Abbreviations

ACLF: acute-on-chronic liver failure
ACLD: advanced chronic liver disease
AFP: alpha fetoprotein
ARLD: alcohol-related liver disease
AUROC: area under the receiver operating curve
BCLC: Barcelona Clinic Liver Cancer classification
CI: confidence interval
CRP: C-reactive protein
CT: computer tomography
CTP: Child-Turcotte-Pugh score
DCR: disease control rate
EASL: European Association for the Study of the Liver
ECOG PS: Eastern Cooperative Oncology Group performance status
HCC: hepatocellular carcinoma
(a)HR: –adjusted hazard ratio
ICB: immune checkpoint blocker
Ig: immunoglobulin
IQR: interquartile range
mRECIST: modified Response Evaluation Criteria in Solid Tumours
MRI: magnetic resonance imaging
NAFLD: non-alcoholic fatty liver disease
NASH: non-alcoholic steatohepatitis
ORR: objective response rate
OS: overall survival
PD-L1: programmed cell death 1 ligand 1
PFS: progression-free survival
(non-)R: –(non-)responder
SD: standard deviation
TTP: time to progression
