# Supplementary figures and images for: Early changes in immunoglobulin G levels during immune checkpoint inhibitor treatment are associated with survival in hepatocellular carcinoma patients
Source: PLoS One. 2023 Apr 7;18(4):e0282680. doi: 10.1371/journal.pone.0282680 (PMC10081755; doi:10.1371/journal.pone.0282680)

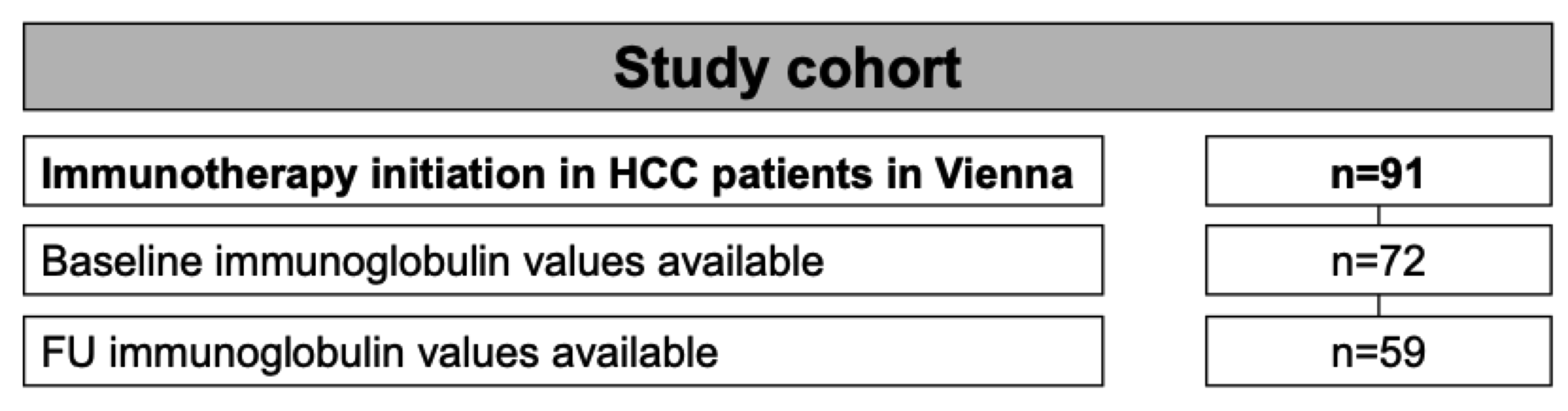

Supplement: S1 Fig — (TIFF) [file pone.0282680.s001.tiff]

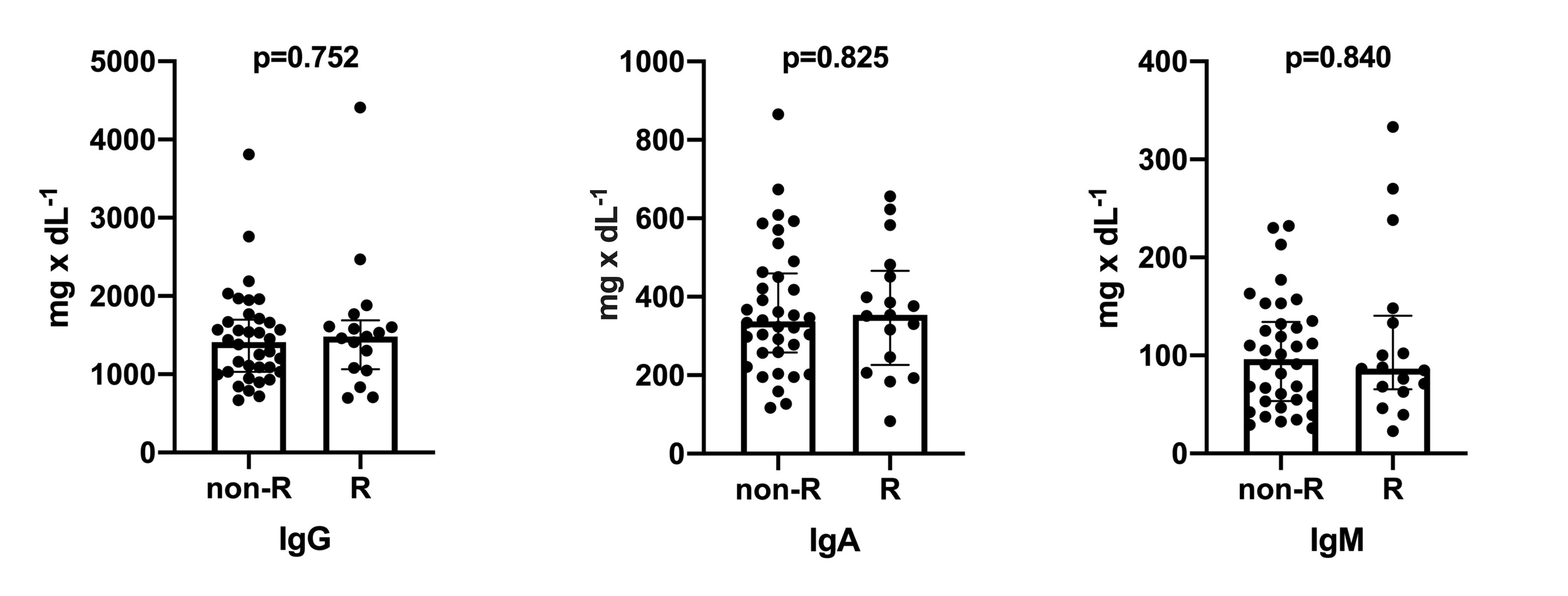

Supplement: S2 Fig — (TIFF) [file pone.0282680.s002.tiff]

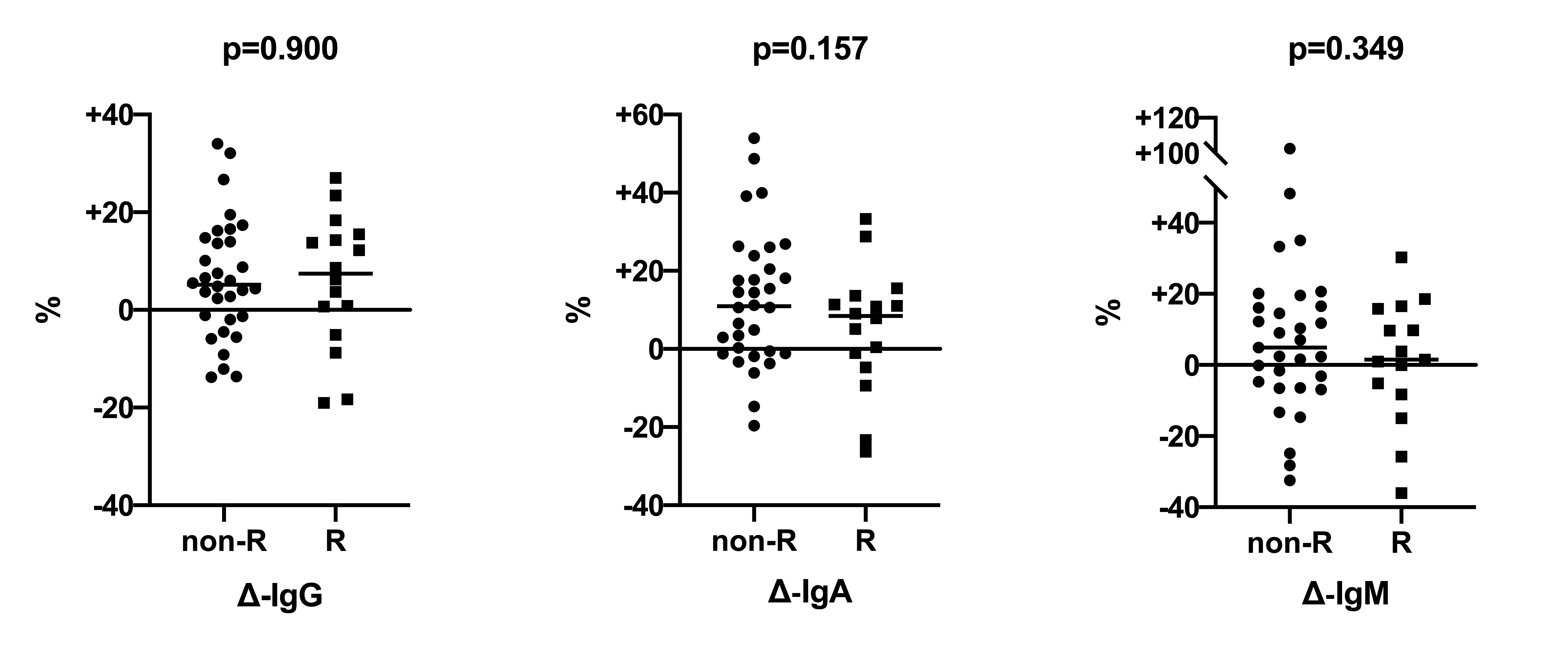

Supplement: S3 Fig — (TIFF) [file pone.0282680.s003.tiff]
