# Supplementary material for: Early changes in immunoglobulin G levels during immune checkpoint inhibitor treatment are associated with survival in hepatocellular carcinoma patients
Source: PLoS One. 2023 Apr 7;18(4):e0282680. doi: 10.1371/journal.pone.0282680 (PMC10081755; doi:10.1371/journal.pone.0282680)
Supplement: S1 Table — (DOCX) [file pone.0282680.s004.docx]

## S1 Table

| *Baseline* | **OS** | | **PFS** | | **TTP** | |
| --- | --- | --- | --- | --- | --- | --- |
|  | **HR (95%CI)** | **p-value** | **HR (95%CI)** | **p-value** | **HR (95%CI)** | **p-value** |
| IgG, per 10, mg x dL^-1^ | 1.00 (0.99-1.00) | 0.667 | 1.00 (0.99-1.00) | 0.294 | 1.00 (0.99-1.00) | 0.144 |
| IgA, per 10, mg x dL^-1^ | 1.01 (0.99-1.03) | 0.244 | 1.00 (0.99-1.02) | 0.716 | 0.99 (0.97-1.02) | 0.531 |
| IgM, per 10, mg x dL^-1^ | 1.00 (0.99-1.01) | 0.727 | 1.00 (0.99-1.01) | 0.693 | 0.99 (0.93-1.05) | 0.735 |
| *Follow-up (week 6)* | **OS** | | **PFS** | | **TTP** | |
|  | **HR (95%CI)** | **p-value** | **HR (95%CI)** | **p-value** | **HR (95%CI)** | **p-value** |
| IgG, per 10, mg x dL^-1^ | 1.00 (0.99-1.01) | 0.767 | 1.00 (0.99-1.00) | 0.737 | 1.00 (0.99-1.00) | 0.277 |
| IgA, per 10, mg x dL^-1^ | 1.01 (0.99-1.03) | 0.143 | 1.01 (0.99-1.02) | 0.654 | 1.00 (0.98-1.02) | 0.947 |
| IgM, per 10, mg x dL^-1^ | 1.00 (0.99-1.02) | 0.610 | 1.00 (0.99-1.01) | 0.900 | 1.00 (0.94-1.05) | 0.897 |

**Supplementary Table 1.** **Univariable Cox regression analyses of immunoglobulin levels at immunotherapy initiation and at week six on overall survival, progression-free survival, and time to progression**

*Abbreviations: Ig immunoglobulin; OS overall survival; PFS progression-free survival; TTP time to progression*
