# Supplementary material for: Early changes in immunoglobulin G levels during immune checkpoint inhibitor treatment are associated with survival in hepatocellular carcinoma patients
Source: PLoS One. 2023 Apr 7;18(4):e0282680. doi: 10.1371/journal.pone.0282680 (PMC10081755; doi:10.1371/journal.pone.0282680)
Supplement: S3 Table — (DOCX) [file pone.0282680.s006.docx]

## S3 Table

| *Patient characteristics* | | **Univariable** | | **Multivariable – first step** | | **Multivariable – last step** | |
| --- | --- | --- | --- | --- | --- | --- | --- |
|  |  | **HR (95%CI)** | **p-value** | **aHR (95%CI)** | **p-value** | **aHR (95%CI)** | **p-value** |
| Age, year | | 1.01 (0.99-1.04) | 0.299 | - | - | - | - |
| Aetiology of liver disease | | | | | | | |
|  | ARLD | 1 | - | - | - | - | - |
|  | Viral | 0.88 (0.35-2.23) | 0.794 | - | - | - | - |
|  | NAFLD | 0.84 (0.33-2.11) | 0.705 | - | - | - | - |
|  | Other | 0.41 (0.14-1.17) | 0.096 | - | - | - | - |
| MVI | | 1.00 (0.49-2.03) | 0.999 | - | - | - | - |
| EHS | | 0.54 (0.26-1.13) | 0.100 | - | - | - | - |
| CTP score | | | | | | | |
|  | A | 1 | - | 1 | - | 1 | - |
|  | B | 2.01 (0.96-4.23) | 0.065 | 1.96 (0.92-4.19) | 0.082 | 1.94 (0.90-4.17) | 0.092 |
|  | C | 5.50 (2.00-15.15) | **<0.001** | 5.92 (2.03-17.28) | **0.001** | 5.07 (1.77-14.50) | **0.002** |
| ECOG PS | | | | | | | |
|  | 0 | 1 | - | - | - | - | - |
|  | ≥1 | 1.60 (0.82-3.12) | 0.166 | - | - | - | - |
| Baseline AFP, per 1000, ng/mL | | 1.03 (1.00-1.05) | **0.025** | 1.02 (0.99-1.05) | 0.127 | - | - |
| Baseline CRP, mg/dL | | 1.23 (1.09-1.38) | **<0.001** | 1.23 (1.08-1.40) | **0.002** | 1.24 (1.09-1.40) | **0.001** |
| Δ-IgG ≥ +14% | | 2.92 (1.46-5.82) | **0.002** | 2.19 (1.01-4.74) | **0.047** | 2.45 (1.18-5.11) | **0.017** |

**Supplementary Table 3.** **Uni- and multivariable Cox regression analyses of prognostic factors for overall survival (OS) (n=59, events n=38)**

*Abbreviations: AFP alpha fetoprotein; ARLD alcohol-related liver disease; CRP C-reactive protein; CTP Child-Turcotte-Pugh score; ECOG PS Eastern Cooperative Oncology Group Performance Status; EHS extrahepatic spread; Ig immunoglobulin; MVI macrovascular invasion; NAFLD non-alcoholic fatty liver disease*
