# Supplementary material for: Early changes in immunoglobulin G levels during immune checkpoint inhibitor treatment are associated with survival in hepatocellular carcinoma patients
Source: PLoS One. 2023 Apr 7;18(4):e0282680. doi: 10.1371/journal.pone.0282680 (PMC10081755; doi:10.1371/journal.pone.0282680)
Supplement: S4 Table — (DOCX) [file pone.0282680.s007.docx]

## S4 Table

| *Patient characteristics* | | **Univariable** | | **Multivariable – first step** | | **Multivariable – last step** | |
| --- | --- | --- | --- | --- | --- | --- | --- |
|  |  | **HR (95%CI)** | **p-value** | **aHR (95%CI)** | **p-value** | **aHR (95%CI)** | **p-value** |
| Age, year | | 1.03 (0.99-1.06) | 0.135 | - | - | - | - |
| Aetiology of liver disease | | | | | | | |
|  | ARLD | 1 | - | - | - | - | - |
|  | Viral | 0.81 (0.23-2.83) | 0.744 | - | - | - | - |
|  | NAFLD | 1.07 (0.31-3.66) | 0.919 | - | - | - | - |
|  | Other | 0.43 (0.10-1.80) | 0.248 | - | - | - | - |
| MVI | | 1.07 (0.44-2.60) | 0.884 | - | - | - | - |
| EHS | | 0.64 (0.26-1.56) | 0.325 | - | - | - | - |
| ECOG PS | | | | | | | |
|  | 0 | 1 | - | - | - | - | - |
|  | ≥1 | 1.62 (0.70-3.74) | 0.257 | - | - | - | - |
| Baseline AFP, per 1000, ng/mL | | 1.03 (1.00-1.06) | **0.035** | 1.02 (0.99-1.05) | 0.187 | - | - |
| Baseline CRP, mg/dL | | 1.25 (1.07-1.44) | **0.004** | 1.24 (1.06-1.45) | **0.007** | 1.22 (1.05-1.42) | **0.009** |
| Δ-IgG ≥ +14% | | 2.82 (1.15-6.91) | **0.023** | 2.05 (0.73-5.75) | 0.171 | 2.50 (0.99-6.33) | 0.052 |

**Supplementary Table 4.** **Uni- and multivariable Cox regression analyses of prognostic factors for overall survival (OS) in patients with preserved liver function at baseline (i.e., Child-Pugh A5-B7) (n=40, events n=24)**

*Abbreviations: AFP alpha fetoprotein; ARLD alcohol-related liver disease; CRP C-reactive protein; CTP Child-Turcotte-Pugh score; ECOG PS Eastern Cooperative Oncology Group Performance Status; EHS extrahepatic spread; Ig immunoglobulin; MVI macrovascular invasion; NAFLD non-alcoholic fatty liver disease*
