# Supplementary material for: Early changes in immunoglobulin G levels during immune checkpoint inhibitor treatment are associated with survival in hepatocellular carcinoma patients
Source: PLoS One. 2023 Apr 7;18(4):e0282680. doi: 10.1371/journal.pone.0282680 (PMC10081755; doi:10.1371/journal.pone.0282680)
Supplement: S5 Table — < +14% groups. (DOCX) [file pone.0282680.s008.docx]

## S5 Table

| *Patient characteristics* | | **Δ-IgG ≥14%**  **n=19** | **Δ-IgG <14%**  **n=40** | **p-value** |
| --- | --- | --- | --- | --- |
| Age, years, mean ± SD | | 66.3±11.9 | 70.0±11.9 | 0.262 |
| Sex, n (%) | | | | |
|  | Male | 14 (74%) | 30 (75%) | 1.000 |
|  | Female | 5 (26%) | 10 (25%) |  |
| Aetiology, n (%) | | | | |
|  | Viral | 7 (37%) | 11 (28%) | 0.629 |
|  | NAFLD | 4 (21%) | 12 (30%) |  |
|  | ARLD | 5 (26%) | 7 (18%) |  |
|  | Other | 3 (16%) | 10 (25%) |  |
| BMI, kg*m^-2^, mean ± SD | | 26.4±4.3 | 27.3±4.5 | 0.438 |
| Prior surgery/ablative therapy, n (%) | | 11 (58%) | 21 (53%) | 0.698 |
| Prior systemic therapy, n (%) | | 4 (21%) | 10 (25%) | 1.000 |
| Cirrhosis, n (%) | | 17 (90%) | 28 (70%) | 0.188 |
| CTP score, points, mean ± SD | | 7.2±1.8 | 6.7±2.1 | 0.336 |
|  | A, n (%) | 7 (37%) | 23 (58%) | 0.331 |
|  | B, n (%) | 9 (47%) | 13 (33%) |  |
|  | C, n (%) | 3 (16%) | 4 (10%) |  |
| Macrovascular invasion, n (%) | | 6 (32%) | 15 (38%) | 0.657 |
| Extrahepatic spread, n (%) | | 9 (47%) | 15 (38%) | 0.471 |
| ECOG PS, n (%) | | | | |
|  | 0 | 8 (42%) | 32 (80%) | **0.006** |
|  | 1 | 9 (47%) | 8 (20%) |  |
|  | 2 | 2 (11%) | - |  |
| BCLC stages, n (%) | | | | |
|  | A | - | 2 (5%) | 0.701 |
|  | B | 4 (21%) | 10 (25%) |  |
|  | C | 12 (63%) | 24 (60%) |  |
|  | D | 3 (16%) | 4 (10%) |  |
| Laboratory parameters, median (IQR) | | | | |
|  | IgA, mg/dL | 321 (233-451) | 350 (264-525) | 0.574 |
|  | IgM, mg/dL | 81 (61-133) | 101 (48-156) | 0.386 |
|  | CRP, mg/dL | 1.5 (0.6-3.0) | 0.6 (0.4-1.8) | 0.092 |
|  | AFP, ng/mL | 154 (6-3311) | 32 (4-693) | 0.146 |

**Supplementary Table 5. Comparison of baseline characteristics between the Δ-IgG ≥ +14% vs. < +14% groups**

*Abbreviations: AFP alpha fetoprotein; ARLD alcohol-related liver disease; BCLC Barcelona Clinic Liver Cancer; BMI body mass index; CRP C-reactive protein; CTP Child-Turcotte-Pugh score; ECOG PS Eastern Cooperative Oncology Group Performance Status; Ig immunoglobulin; IQR interquartile range; NAFLD non-alcoholic fatty liver disease; SD standard deviation*
