# Supplementary material for: Early changes in immunoglobulin G levels during immune checkpoint inhibitor treatment are associated with survival in hepatocellular carcinoma patients
Source: PLoS One. 2023 Apr 7;18(4):e0282680. doi: 10.1371/journal.pone.0282680 (PMC10081755; doi:10.1371/journal.pone.0282680)
Supplement: S6 Table — (DOCX) [file pone.0282680.s009.docx]

## S6 Table

| *Patient characteristics* | | **Univariable** | | **Multivariable – first step** | | **Multivariable – last step** | |
| --- | --- | --- | --- | --- | --- | --- | --- |
|  |  | **HR (95%CI)** | **p-value** | **aHR (95%CI)** | **p-value** | **aHR (95%CI)** | **p-value** |
| Age, year | | 0.99 (0.97-1.02) | 0.711 | - | - | - | - |
| Aetiology of liver disease | | | | | | | |
|  | ARLD | 1 | - | - | - | - | - |
|  | Viral | 2.02 (0.65-6.30) | 0.226 | - | - | - | - |
|  | NAFLD | 1.69 (0.52-5.51) | 0.385 | - | - | - | - |
|  | Other | 0.98 (0.28-3.50) | 0.980 | - | - | - | - |
| MVI | | 0.44 (0.20-0.99) | **0.046** | 0.48 (0.18-1.28) | 0.145 | - | - |
| EHS | | 1.42 (0.69-2.92) | 0.340 | - | - | - | - |
| ECOG PS | | | | | | | |
|  | 0 | 1 | - | 1 | - | - | - |
|  | ≥1 | 2.06 (0.98-4.31) | 0.056 | 1.95 (0.76-5.00) | 0.164 | - | - |
| Baseline AFP, per 1000, ng/mL | | 1.02 (0.99-1.04) | 0.194 | - | - | - | - |
| Baseline CRP, mg/dL | | 1.15 (1.02-1.30) | **0.026** | 1.09 (0.88-1.35) | 0.419 | - | - |
| Δ-IgG | | 1.05 (1.01-1.08) | **0.006** | 1.03 (0.99-1.02) | 0.095 | 1.05 (1.02-1.09) | **0.003** |
| Δ-IgA | | 1.01 (0.98-1.03) | 0.637 | - | - | - | - |
| Δ-IgM | | 1.01 (1.00-1.02) | **0.021** | 1.00 (0.99-1.02) | 0.637 | - | - |

**Supplementary Table 6.** **Uni- and multivariable Cox regression analyses of prognostic factors for progression-free survival (PFS) in patients with preserved liver function at baseline (i.e., Child-Pugh A5-B7) (n=40, events n=32)**

*Abbreviations: AFP alpha fetoprotein; ARLD alcohol-related liver disease; CRP C-reactive protein; CTP Child-Turcotte-Pugh score; ECOG PS Eastern Cooperative Oncology Group Performance Status; EHS extrahepatic spread; Ig immunoglobulin; MVI macrovascular invasion; NAFLD non-alcoholic fatty liver disease*
