# Supplementary material for: Early changes in immunoglobulin G levels during immune checkpoint inhibitor treatment are associated with survival in hepatocellular carcinoma patients
Source: PLoS One. 2023 Apr 7;18(4):e0282680. doi: 10.1371/journal.pone.0282680 (PMC10081755; doi:10.1371/journal.pone.0282680)
Supplement: S7 Table — (DOCX) [file pone.0282680.s010.docx]

## S7 Table

| *Patient characteristics* | | **Univariable** | | **Multivariable – first and last step** | |
| --- | --- | --- | --- | --- | --- |
|  |  | **HR (95%CI)** | **p-value** | **aHR (95%CI)** | **p-value** |
| Age, year | | 1.00 (0.96-1.04) | 0.958 | - | - |
| Aetiology of liver disease | | | | | |
|  | ARLD | 1 | - | - | - |
|  | Viral | 1.70 (0.44-6.52) | 0.439 | - | - |
|  | NAFLD | 1.62 (0.42-6.32) | 0.484 | - | - |
|  | Other | 1.29 (0.28-5.88) | 0.741 | - | - |
| MVI | | 0.29 (0.10-0.83) | **0.021** | 0.36 (0.12-1.05) | 0.062 |
| EHS | | 1.74 (0.75-4.02) | 0.197 | - | - |
| CTP score | | | | | |
|  | A | 1 | - | - | - |
|  | B | 0.74 (0.25-2.22) | 0.596 | - | - |
| ECOG PS | | | | | |
|  | 0 | 1 | - | - | - |
|  | ≥1 | 1.61 (0.66-3.91) | 0.293 | - | - |
| Baseline AFP, per 1000, ng/mL | | 1.00 (0.96-1.05) | 0.858 | - | - |
| Baseline CRP, mg/dL | | 0.98 (0.75-1.29) | 0.900 | - | - |
| Δ-IgG, % | | 1.04 (1.01-1.08) | **0.027** | 1.04 (0.99-1.08) | 0.089 |
| Δ-IgA, % | | 0.99 (0.97-1.02) | 0.652 | - | - |
| Δ-IgM, % | | 1.01 (0.98-1.03) | 0.705 | - | - |

**Supplementary Table 7.** **Uni- and multivariable Cox regression analyses of prognostic factors for time to progression (TTP) in patients with preserved liver function at baseline (i.e., Child-Pugh A5-B7) (n=34, events n=23)**

*Abbreviations: AFP alpha fetoprotein; ARLD alcohol-related liver disease; CRP C-reactive protein; CTP Child-Turcotte-Pugh score; ECOG PS Eastern Cooperative Oncology Group Performance Status; EHS extrahepatic spread; Ig immunoglobulin; MVI macrovascular invasion; NAFLD non-alcoholic fatty liver disease*
