# Supplementary material for: Early changes in immunoglobulin G levels during immune checkpoint inhibitor treatment are associated with survival in hepatocellular carcinoma patients
Source: PLoS One. 2023 Apr 7;18(4):e0282680. doi: 10.1371/journal.pone.0282680 (PMC10081755; doi:10.1371/journal.pone.0282680)
Supplement: S8 Table — (DOCX) [file pone.0282680.s011.docx]

## S8 Table

| *Radiological response* | | **Δ-IgG ≥ +14%** | **Δ-IgG < +14%** | **p-value** |
| --- | --- | --- | --- | --- |
| Disease control rate, n (%) | | | | |
|  | Yes | 8 (62%) | 26 (74%) | 0.480 |
|  | No | 5 (38%) | 9 (26%) |  |
| Overall response rate, n (%) | | | | |
|  | Yes | 5 (38%) | 11 (31%) | 0.735 |
|  | No | 8 (62%) | 24 (69%) |  |
| Radiological response according to mRECIST, n (%) | | | | |
| CR or PR | | 5 (38%) | 11 (31%) | 0.437 |
| SD | | 3 (24%) | 15 (43%) |  |
| PD | | 5 (38%) | 9 (26%) |  |

**Supplementary Table 8.** **Association of Δ-immunoglobulin G with best radiological response**

*Abbreviations: CR complete response; DCR disease control rate; Ig immunoglobulin; ORR overall response rate; PD progressive disease; PR partial response; SD stable disease*
