# Supplementary material for: Early changes in immunoglobulin G levels during immune checkpoint inhibitor treatment are associated with survival in hepatocellular carcinoma patients
Source: PLoS One. 2023 Apr 7;18(4):e0282680. doi: 10.1371/journal.pone.0282680 (PMC10081755; doi:10.1371/journal.pone.0282680)
Supplement: S2 File — (PDF) [file pone.0282680.s013.pdf]

Early changes in immunoglobulin G levels during immune checkpoint inhibitor treatment are associated with survival in hepatocellular carcinoma patients

Aim: Evaluation of changes in immunoglobulin levels in patients with hepatocellular carcinoma receiving immunotherapy

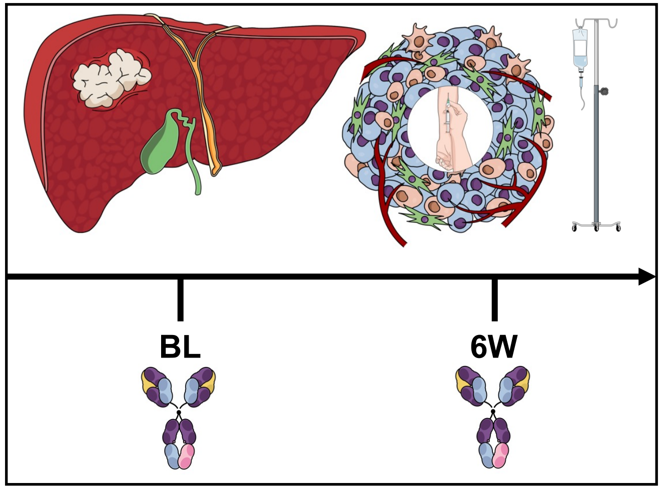

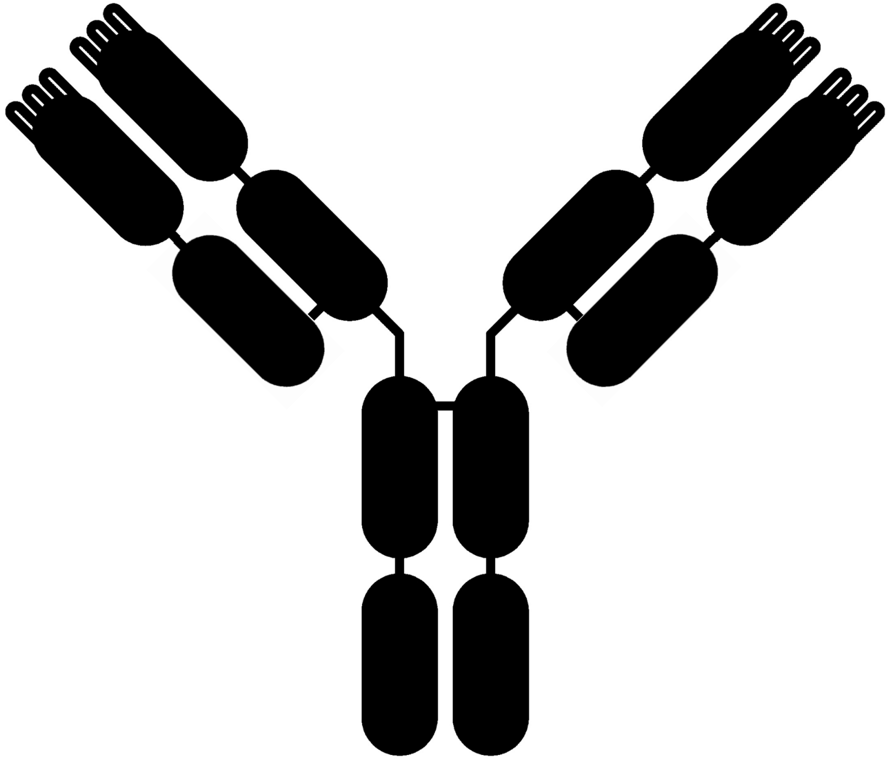

Δ-IgG was independently associated with OS, PFS and TTP in multivariable Cox regression analysis

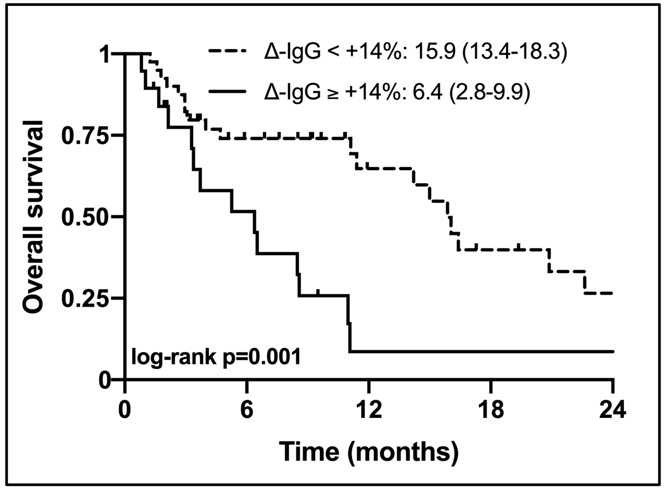

Δ-IgG at week 6 (cut-off ≥14%) after immunotherapy initiation is a negative prognostic marker in patients with hepatocellular carcinoma
